# Pediatric Acute Promyelocytic Leukemia Presenting to the Emergency Department as Refusal to Ambulate

**DOI:** 10.1155/2018/5241425

**Published:** 2018-06-12

**Authors:** Kevin R. Schwartz, Jennifer M. Hanson, Alison M. Friedmann

**Affiliations:** ^1^Departments of Emergency Medicine and Pediatrics, Massachusetts General Hospital, Harvard Medical School, USA; ^2^Department of Pediatrics, Massachusetts General Hospital, Harvard Medical School, USA

## Abstract

A previously healthy 10-year-old girl presented to the emergency department (ED) with a headache and vomiting which resolved with oral NSAIDs. The patient returned two days later unable to ambulate with mental slowing and lower extremity bruising. Labs demonstrated marked leukocytosis, severe anemia and thrombocytopenia, and disseminated intravascular coagulation (DIC). Brain MRI showed multiple intracranial hemorrhages. A peripheral blood smear demonstrated blasts with many Auer rods. A diagnosis of acute promyelocytic leukemia (APL) was made and therapy including all-transretinoic acid (ATRA) was initiated. Neurologic status returned to baseline within 1 week in the pediatric intensive care unit.

## 1. Introduction

Acute promyelocytic leukemia (APL) is a rare form of acute myeloid leukemia (AML) representing less than 10% of* de novo* pediatric AMLm [1]. While APL is a highly curable malignancy with more than 80% of patients surviving long term, up to 10% of patients die early in the course of their disease due to hemorrhage, primarily intracranial [2]. The early initiation of appropriate therapy when APL is suspected has been demonstrated to reduce hemorrhagic complications and early death [3]. As such, it is critical for emergency medicine providers to be familiar with the presentation, diagnosis, and initial treatment of APL [4].

## 2. Case Presentation

A previously healthy 10-year-old Asian girl presented to the emergency department with headache, vomiting, and one week of mild nonproductive cough. Her headache started the evening prior to presentation, was gradual in onset and frontotemporal in location, and improved with acetaminophen but subsequently woke her from sleep. It was accompanied by two episodes of emesis. On presentation to the ED, the patient described her headache pain as 3 out of 10 in severity. She denied photophobia, had no further nausea, and denied abdominal pain. She reported that the headache worsened with standing and improved with lying down. Review of systems was significant only for pallor.

The patient was otherwise healthy with no prior medical issues and taking no regular medications. She was fully vaccinated and had no known allergies. Her family history was significant for frequent headaches in her mother and maternal aunt. She was living with her parents and brother and attending 4^th^ grade.

Vital signs demonstrated blood pressure 111/56, pulse 104, temperature 37.1°C, respiratory rate 22, and oxygen saturation 100% on room air. Initial exam revealed a well-appearing female and was unremarkable including a normal fundoscopic exam and a normal complete neurologic exam.

The patient received ibuprofen and oral rehydration and her headache further improved. A presumptive diagnosis of migraine headache was made and was discharged with primary care follow-up the following day.

Two days after her initial emergency department visit, the patient returned to the ED with worsening headache, myalgia, subjective fever, and diffuse weakness. The patient's mother reported that the patient was unable to stand or walk and as a result her mother had been carrying her, including to and from the bathroom. The patient endorsed nausea but no further vomiting.

Vital signs demonstrated blood pressure 105/49, pulse 123, temperature 36.9°C, respiratory rate 30, and oxygenation saturation of 97% on room air. On exam, the patient was moderately ill appearing, lying in bed responding slowly to questions. Her lips were noted to be cracked and with some oozing blood. No oral lesions were noted in the mouth. Pupils were equal, round, and sluggishly reactive bilaterally. Neck was supple with no adenopathy noted. Cardiovascularly, she was noted to be tachycardic with a regular rhythm and II/VI flow murmur. Her respiratory exam was normal. Her abdominal exam was benign with no organomegaly. Neurologically the patient was noted to be slow to respond to questions and moving slowly but without focal deficits. She was, however, unable to walk without assistance. Skin exam revealed diffuse ecchymoses on the lower extremities bilaterally.

Laboratory studies were ordered along with a rapid brain MRI, and pediatric neurology was consulted.

**Figure 1:**
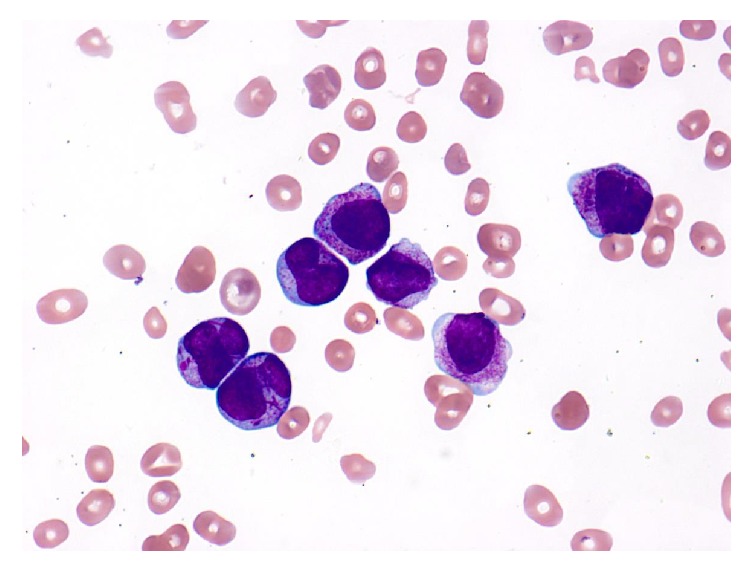
Peripheral blood smear demonstrating many primitive cells with round and lobated nuclei and moderately abundant cytoplasm with numerous cytoplasmic granules. Auer rods were readily identified; some cells had multiple Auer rods. A classic appearance for APL.

Laboratory results were as follows: hemoglobin 2.6 g/dL, hematocrit 8.3%, platelets 10K/uL, and WBC 60.5K/uL with 83% blasts in the differential. CRP was 15.7 mg/L, and ESR was 125 mm/h. Electrolytes showed sodium 139 mmol/L, potassium 4.0 mmol/L, chloride 100 mmol/L, carbon dioxide 24 mmol/L, BUN 11mg/dL, creatinine 0.56 mg/dL, glucose 129, magnesium 2.5mg/dL, and phosphorus 3.6 mg/dL. LDH was 359 U/L, uric acid was 2.3mg/dL. PTT was 29.4 seconds, PT was 19.5 seconds, and INR was 1.7. Fibrinogen was 117 mg/dL and D-dimer was >10,000 ng/mL. Review of peripheral blood smear (Figure 1) demonstrated many primitive cells with round and lobated nuclei, numerous cytoplasmic granules with Auer rods readily identified, and some cells with multiple Auer rods.

**Figure 2:**
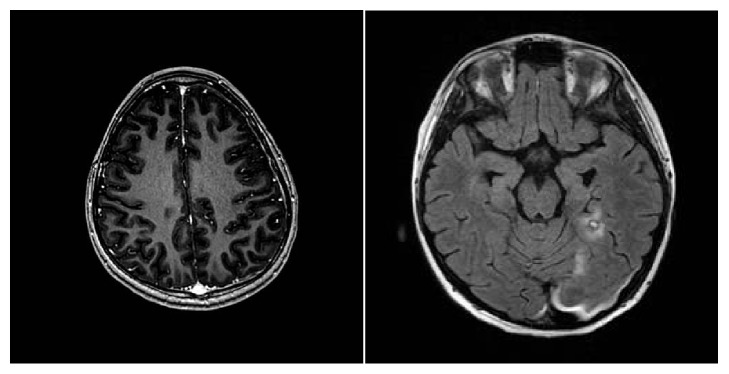
MRI Brain FLAIR images demonstrating multicompartmental hemorrhages and leptomeningeal enhancement.

Rapid MRI Brain (Figure 2) was obtained which demonstrated leptomeningeal enhancement in the supratentorial parenchyma suggestive of leptomeningeal carcinomatosis, a hemorrhagic lesion in the corpus callosum, multiple subdural hematomas with mild mass effect, and petechial hemorrhages throughout the brain.

Pediatric Oncology was consulted and treatment initiated emergently with ATRA, dexamethasone, allopurinol, cefepime, and blood products including packed red blood cells, platelets, and cryoprecipitate. The patient was subsequently admitted to the pediatric intensive care unit.

### 2.1. Hospital Course

In the pediatric ICU the patient received several transfusions with platelets, cryoprecipitate, and fresh frozen plasma to manage DIC. She was continued on an induction course of chemotherapy including ATRA, dexamethasone, idarubicin, and arsenic trioxide. Molecular analysis of the peripheral blood was positive for PML-RARA, confirming the diagnosis of APL. After the coagulopathy improved and the patient stabilized, lumbar puncture was performed with administration of intrathecal chemotherapy. The cerebrospinal fluid was notable for the presence of leukemia cells, confirming the involvement of the central nervous system. The patient's encephalopathy gradually improved and she returned to her baseline mental status by day 7 of treatment. The patient was noted to have elevated opening pressure on lumbar punctures, which worsened over the course of induction, attributed to pseudotumor cerebri secondary to ATRA.

Upon completion of the first 28 days of induction therapy, repeat lumbar puncture and bone marrow studies demonstrated no morphologic evidence of acute promyelocytic leukemia, consistent with remission.

## 3. Discussion

There are a total of 600-800 new cases of APL diagnosed each year in the United States, the majority of which occur in adults [5]. The peak age of presentation within pediatrics is 9-12 years old and there is a slight female predominance [1, 6]. APL, the French-American-British (FAB) M3 subtype of AML, results from clonal proliferation of myeloid lineage cells that are arrested at the promyelocyte stage. This maturational arrest results from a characteristic translocation between the PML gene on chromosome 15 and the Retinoic Acid Receptor-alpha (RARA) gene on chromosome 17, t(15;17)(q22;q21.1). This translocation results in production of the fusion protein PML-RARA which represses nuclear gene transcription, arresting myelocytes at the promyelocytic stage of maturation [2, 7].

On peripheral blood smear, APL blasts have a characteristic appearance with abundant cytoplasmic granules, bilobed nuclei, and prominent Auer rods (Figure 1). While up to one-third of pediatric patients may not demonstrate this morphology, when these cells are indeed present on peripheral blood smear in the proper clinical context, a diagnosis of APL is extremely likely [2, 7]. Given the high up-front mortality rate, treatment should not be delayed pending molecular or immunophenotyping studies and should be initiated as soon as APL is suspected based on clinical presentation and/or the peripheral blood smear [8].

Children with APL usually present with the signs and symptoms of cytopenias common to other leukemias. Fatigue, pallor, bruising, and fevers are frequent complaints [6]. Extramedullary diseases such as hepatomegaly, splenomegaly, and lymphadenopathy are seen less commonly in APL compared with other subtypes of AML, and CNS leukemia is rare [1, 9].

The central distinguishing feature of APL, as compared to other forms of leukemia, is its propensity to cause disseminated intravascular coagulopathy (DIC). 80-90% of patients with APL will have evidence of bleeding diathesis on initial presentation [4, 7, 10]. DIC is characterized by prolonged PT/INR and PTT, low fibrinogen, elevated D-dimer, elevated fibrin split products, and thrombocytopenia. Bleeding is consequently a common presentation of APL, with intracranial hemorrhage representing the primary site of severe bleeding in more than two-thirds of cases [10]. Aggressive management of DIC as well as initiation of ATRA as soon as APL is suspected is critical to reduce the risk of death from intracranial hemorrhage, the most common cause of early death in APL patients [8].

All-transretinoic acid (ATRA), a vitamin A derivative, binds to the RARA site of the PML-RARA fusion protein in APL cells and allows the leukemic promyelocytes to differentiate into mature granulocytes. This leads to a rapid improvement in coagulopathies [3, 4]. ATRA should be given as soon as APL is suspected in the emergency department and has been associated with decreased rates of early hemorrhagic death and decreased requirements for blood product support [3, 11]. Delaying ATRA administration has been associated with increased incidence of early hemorrhagic death [12]. Recommended dosing for ATRA is 25mg/m^2^ daily and it is only available orally. For pediatric patients unable to swallow capsules, either can ATRA capsules be softened in warm milk and swallowed orally in soft food or capsule contents can be drawn up in a syringe from the softened capsule and administered via NG tube [2, 13].

In conjunction with therapy for suspected APL with ATRA, aggressive therapy of DIC should be undertaken. Fresh frozen plasma and/or cryoprecipitate should be transfused to maintain fibrinogen levels over 150 g/dL and platelets should be transfused to maintain platelet counts over 50 x10^9^/liter. Heparin has not been demonstrated to have a clear benefit and is not recommended for DIC related to APL [1, 4].

The overall prognosis of APL is excellent, with more than 90% of patients achieving complete remission and 5-year overall survival rates in excess of 80%. On average, coagulopathy in APL corrects within a median time of 4 days after the initiation of ATRA [3]. In contrast to other forms of AML, for patients with standard risk APL, cure may be achieved with ATRA and Arsenic Trioxide (ATO) alone [14]. Treatment of high risk disease generally incorporates the addition of anthracycline to an ATO and ATRA based regimen.

While ATRA avoids many of the side effects associated with traditional cytotoxic drugs, it does have some significant associated adverse reactions including pseudotumor cerebri and differentiation syndrome. ATRA toxicity, including pseudotumor cerebri, appears to be more common in children as compared to adults [15]. Differentiation syndrome, characterized by fever, respiratory distress, pleural and pericardial effusions, hypotension, and/or renal failure, results from the excessive inflammatory response and cytokine release which can result from inducing maturation of promyelocytes with ATRA. Treatment of this entity consists of discontinuation of ATRA, administering dexamethasone and providing cardiorespiratory support [2].

## 4. Conclusion

APL is a rare disease in children, and its characteristic presentation of typical leukemia signs and symptoms accompanied by DIC/bleeding and its characteristic morphology on peripheral blood smear are important to recognize because initiation of disease-targeted therapy is associated with decreased mortality from hemorrhagic complications. Pediatric emergency providers should have a high index of suspicion for APL in pediatric patients presenting with pancytopenia accompanied by DIC. ATRA therapy, in consultation with a pediatric oncologist, accompanied by platelet and fibrinogen replacement to manage DIC should be initiated as soon as APL is suspected based on clinical presentation and/or peripheral blood smear.
